# Prevalence of clinically captured and confirmed malaria among HIV seropositve clinic attendants in five hospitals in Ghana

**DOI:** 10.1186/1475-2875-12-382

**Published:** 2013-10-30

**Authors:** Dennis Adu-Gyasi, Caterina I Fanello, Frank Baiden, John DH Porter, Dan Korbel, George Adjei, Emmanuel Mahama, Alexander Manu, Kwaku Poku Asante, Sam Newton, Seth Owusu-Agyei

**Affiliations:** 1 Kintampo Health Research Centre, P. O. Box 200, Kintampo North, Ghana; 2 Mahidol-Oxford Research Unit, Faculty of Tropical Medicine, Mahidol University, Bangkok, Thailand; 3 London School of Hygiene and Tropical Medicine, Keppel Street, London WC1E 7HT, UK; 4 The Wellcome Trust Gibbs Building, 215 Euston Road, London NW1 2BE, UK

## Abstract

**Background:**

Malaria is associated with an increase in HIV viral load and a fall in CD4-cell count. Conversely, HIV infection disrupts the acquired immune responses to malaria and the efficacy of antimalarial drugs. This study was carried out in five Ghanaian hospitals to estimate the prevalence of clinically confirmed malaria among HIV patients by evaluating their hospital records.

**Methods:**

This retrospective descriptive cross sectional study reviewed and collected data on malaria, using Case Record Forms from HIV patients’ folders in five hospitals in Ghana.

**Results:**

There were 933 patients records made up of 272 (29.2%) males and 661 (70.8%) females. Majority of the patients were aged between 21–40 (63.6%) years and the rest were between the ages 1–20 (2.8%) years, 41–60 (31.6%) years and 61–80 (2.1%) years of age.

A total of 38.1% (355/933) of the patients were clinically suspected of having clinical malaria. Of these 339 (95.5%) were referred to the laboratory for confirmation of the diagnosis of malaria. Only 4.4% (15/339) of patients tested were confirmed as cases of malaria among the patients that were clinically suspected of having malaria and subsequently confirmed. Fever, was not significantly associated with a confirmed diagnosis of malaria [OR = 3.11, 95% CI: (0.63, 15.37), P = 0.142].

**Conclusions:**

There was a 4.4% prevalence of confirmed malaria and 38.1% of presumptively diagnosed malaria from the case records of HIV patients from the selected hospitals in Ghana.

## Background

Africa has a heavy burden of HIV and malaria infections [1,2], two diseases which are the common infections in sub-Saharan Africa [1]. Over 200–500 million episodes of malaria occur yearly worldwide [3,4] and malaria remains the first cause of loss of days of healthy life in Ghana [5].

Malaria could have possible effects on HIV acquisition, disease progression, and response to therapy [6]. There may be a temporary increase in HIV-RNA and a decrease in CD4-T-lymphocyte count due to other infections as well as to malaria infections.

Malaria is known to be associated with an increase in viral load as noted in a study by Chalwe et al. in Zambia where HIV infected individuals with malaria had significant increase in viral load [7] and a fall in CD4-cell count [8], which could pose a potential threat in the clinical course of people with HIV infection [9]. This phenomenon is more severe in HIV infected adults [10]. Conversely, HIV infection could also disrupt the acquired immune response to malaria; affect the incidence, the frequency and severity of malaria and the efficacy of anti-malarial drugs [11-13].

There are concerns that in malaria endemic areas where HIV prevalence is high interactions with antiretroviral therapy (ART) especially protease inhibitors could affect the utilization of newly introduced artemisinin-based combination therapy (ACT), though this finding was established in a small study [14]. In some cases administration of anti-malarial with anti-retroviral drugs have led to various levels of toxicity due to some effects of HIV-specific factors and drug interactions [15,16].

Ghana, with an estimated population of nearly 24 million, has an estimated 336,000 people living with HIV [17] with considerable variations in various regions of the country [18]. Nearly all regions of Ghana are malaria-endemic with transmission being all year-round. Significant variations however exist in the months of peak incidence of malaria transmission. The crude parasite rates in Ghanaian general population range from 10-70%, with *Plasmodium falciparum* accounting for 90-98% of all infections [19]. The average prevalence of malaria in all age cohorts was given as 58% in a study by Owusu-Agyei et al. conducted in the Kintampo North and South Districts of the Brong-Ahafo Region of Ghana in 2009 [20].

Due to the fact that statistics for the prevalence of malaria among HIV patients is not readily available it became necessary to carry out this study in five hospitals within the Brong-Ahafo and Ashanti Regions of Ghana to establish the prevalence of confirmed malaria using case records of HIV patients registered in the hospitals to receive HIV management and care.

## Methods

### Study clinics/sites

**Figure 1:**
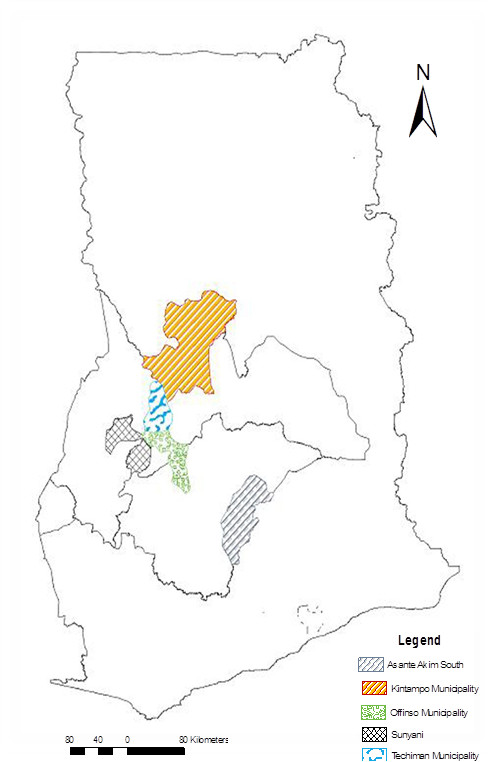
The map of Ghana with the regions and towns of the health facilities that were used for the review were selected.

The facilities used for the study were the Kintampo Municipal Hospital in the Kintampo North Municipality, the Holy Family Hospital in the Techiman Municipality and the Sunyani Regional Hospital all in the Brong-Ahafo Region. Other facilities were used in the Ashanti Region of Ghana and these were St Patrick’s Hospital in the Offinso South Municipality and the Agogo Presbyterian Hospital, in the Asante Akim District. These health facilities were selected for the study because they offered HIV/AIDS management clinics and services (Figure 1) and played significant roles in the management of nearly 35% of HIV patients within the two regions of Ghana [18]. Information on HIV patients from the selected sites is representative of the larger population of HIV patients, their management practices and record keeping are similar to the other facilities in the public hospitals in the other regions of Ghana [21]. Scheduled follow-up visits of patients were made based on patient’s severity of illness and also patient’s records of adherence to management therapy. This was found to be a maximum of every two months. The data collection was carried out in July 2010.

### Study design and procedure

**Figure 2:**
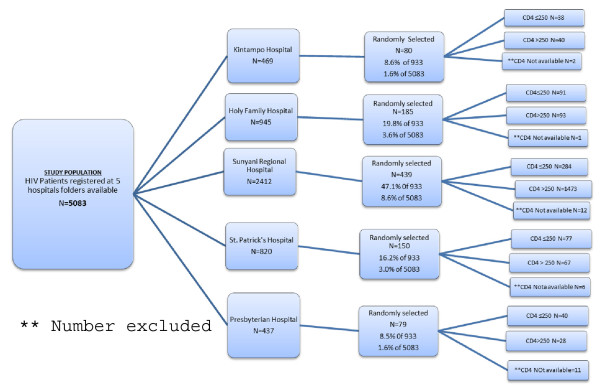
Flow chart for the selection of folders for the malaria in HIV study.

The study was a retrospective and descriptive cross-sectional study. Hospital records of registered seropositive patients attending HIV clinics, from five HIV management sites within Ghana Health Service were selected and randomly reviewed to collect data on malaria cases and HIV, for a one year period following the patient’s registration in each facility. Patients’ registration numbers were used in each facility to randomly select their hospital folders to start the selection process for the review. Total number of folders selected from each facility was based on the total registered patients in each facility based on their date of attendance and how they could potentially contribute to the study (Figure 2).

### Definitions used at data collection

**Malaria:** A case of malaria needed to have been confirmed by the laboratory. A laboratory confirmed cases of malaria was based on either a blood slide reading or the use of rapid diagnostic test (RDT) kits.

**Episode:** An episode of malaria was defined as a reported case from a patient who was examined and found to be indeed a case of malaria and treated.

### Data collection

Data collected from the patients’ folders included; the dates of registration in each facility, patient’s age, sex and socio-demographic information. Other information collected were information on fever and episodes of malaria as well as confirmed diagnosis of malaria. A case record form (CRF) was designed for extraction of the data based on the template used at the HIV management clinics. The category of patients’ folders excluded from review were those who had defaulted on their schedule visits for three or more consecutive times. Data of such individuals were excluded because it would have affected the overall quality of the data and the generalizability of the data.

### HIV diagnosis

HIV diagnosis in each of the facilities was carried out by antibody testing using either First Response HIV 1–2, Rapid HIV Test kit (Premier Medical Corporation Limited, India) or Determine HIV-1/2 test kit (Abbott Laboratories, USA). The test was further confirmed with Ora Quick Rapid HIV-1/2 Antibody Test (OraSure Technologies, Inc, USA). An immunoblot assay, Innolia HIV 1&2 (Innogenetics, N.V. Belgium) was used for clarification of discordant results [21] both at the Tertiary hospitals and the Public Health Reference Laboratories.

### Malaria diagnosis

Malaria cases which were included were based on treated malaria cases either with or without laboratory diagnosis. In the hospitals, blood slide reading for malaria diagnosis was made as follows. A thick blood film was made on a microscope slide and stained with 10% Giemsa for ten (10) minutes. Parasite diagnosis and quantification, using a light microscope, was made using the plus system by WHO (“+”, “++”, “+++”, and “++++”). A standard operating procedure (SOP) was developed and used across the sites. In some laboratories Rapid Diagnostic Test (RDT) kits were used to diagnose malaria. Data for the proportions of laboratory confirmations made with either blood slide reading or RDT were however not available.

To minimize selection bias, an equal proportion of patients’ folders of HIV patients registered in each of the five selected facilities were reviewed.

### Ethics statement

Data was collected from each of the HIV treatment centres in accordance with Good Clinical Practices (GCP). Ethical approvals for the study were given by the Ghana Health Service Ethical Review Committee, the Kintampo Health Research Centre Institutional Ethics Considering that the study involved secondary data analyses, an application was made for ethical approvals from the ethics committees for a waiver for each individual patient to give informed consent. Permission was also sought from the National AIDS/STI Control Programme, the Ghana AIDS Commission and the Managers of the various facilities selected for the study.

### Quality control

Ten per cent (10%) of all folders reviewed in each facility were verified and checked using the patients’ folders and the CRFs that had been filled. All CRFs were checked on-site to ensure missing data or blanks were not the result of errors made during data collection but actually existed as blanks in patients’ folders.

### Data management and strategy for data analysis

Microsoft Access 2007 data management software and Stata 11 software (Stata Corporation, TX USA) were used for data entry and data analyses respectively. Verification and consistency checks were performed with reference to the Case Record Forms. Multivariate logistic regression models were used to adjust for probable confounding factors.

## Results

### Demographic and social characteristics

A total of 933 patients’ folders were reviewed from the five hospitals. The Presbyterian Hospital in Agogo contributed (8.5%), St. Patrick’s Hospital in Offinso (16.1%), Kintampo Municipal Hospital (8.6%), Holy Family Hospital, Techiman (19.8%) and Sunyani Regional Hospital (47.1%).

Of the 933 patients, 272 (29.2%) were males and 661 (70.8%) were females. Patients enrolled into the study and with no missing records of age were made of ages 1–20 (2.8%) years, 41–60 (31.6%) years, 61–80 (2.1%) years. Majority of them were aged between 21–40 (63.6%) years. Included in the folders reviewed were records of 1.2% (11/933) children with their ages less than 10 years.

Data of the patients’ weights at enrolment were present in 834 (89.4%) folders and missing in 99 (10.6%). Records for both age and weight of patients at enrolment, were available for 780 patients (83.6%). The mean weight (S.D) at enrolment was 53.0 kg (12.0) for males and 51.0 kg (10.0) for females (Table 1). All missing records were excluded during data analyses.

**Table 1:** Summary of age and weight of patients by sex

**Age group (y)**	**Male**	**Mean weight (kg) at registration (SD)**	**Female**	**Mean weight (kg) at registration (SD)**
	**n (%)**		**n (%)**	
<21	12 (5.4)	26.7 (19.4)	8 (1.4)	50.6 (9.4)
21-40	90 (40.5)	54.3 (8.8)	404 (72.4)	51.5 (9.8)
41-60	114 (51.4)	54.8 (10.2)	135 (24.2)	49.8 (10.6)
61-80	6 (2.7)	51.1 (8.7)	11 (2.0)	48.3 (8.7)
Total (N = 780)	222 (28.5)	53.0 (12.0)	558 (71.5)	51.0 (10.0)

Records not available for both initial weight and age: 153/933.

Overall, 627 (67.2%) of the 933 patients were on antiretroviral therapy in the period under review and 89% (558/627) were compliant in their drug-use. There were no information about the HIV-RNA of the patients.

### Malaria prevalence

In the 933 folders reviewed, 355 (38.1%) reported a diagnosis of clinical malaria out of which 339 (95.5%) were referred to the laboratory for confirmation. Only 15 patients were confirmed as positive, representing 4.4%. Nevertheless, all 355 patients were given anti-malarial treatment. There was no significant association between confirmed malaria cases and age groups of patients (p = 0.499) neither was there any association between diagnosis and weights (p = 0.386) at enrolment.

### Malaria and ART status

Of the 15 confirmed malaria cases, 13 (86.7%) were on ART; and the remaining 2 (13.3%) were not on ART. There was no significant difference between patients who were on ART and had malaria and those not on ART but had malaria [OR = 2.42, 95% CI: (0.53, 11.02), P = 0.237]. However, the number of cases remains few and any interpretation should be made with caution. However, considering malaria diagnosed presumptively, expectedly 248/614 (40.4%) of patients on ART were diagnosed with clinical malaria as compared to 92/304 (30.3%) of those who were not on ART but had clinical malaria [OR = 1.56, 95% CI: (1.16, 2.10), P = 0.003]. Malaria was over-diagnosed based on the presence of fever (OR = 4.11, 95% CI: [2.83, 5.96], P < 0.001).

## Discussion

Following the review of the clinical records, the study recorded 4.4% (95% CI: 2.2, 6.6) prevalence of laboratory confirmed malaria and 38.1% (95% CI: 34.9, 41.2) of malaria diagnosed purely by clinical signs and symptoms among HIV patients in the two regions of Ghana. This prevalence of confirmed malaria is consistent with the prevalence of 5.8% of HIV-Malaria co-infection recorded by Tshikuka *et al*. [22] and clinically captured malaria compared to records established by Owusu-Agyei *et al.* Though it has been established in this study that the practice of presumptive diagnosis of malaria is high 38.1%, one would have still expected a high prevalence of confirmed malaria among a population with known immune suppression [23-25]. A low prevalence of malaria compared to the prevalence obtained by presumptive diagnosis among HIV patients on co-trimoxazole prophylaxis (COT) could confirm the protective effect of COT against malaria [10]. One interesting finding was that, there was no significant difference in the prevalence of malaria among patients who were on ART and those who were not. However, there was an increased risk of being diagnosed with confirmed cases of malaria if one was on ART, though this was not statistically significant [OR = 2.42, 95% CI: (0.53, 11.02), P = 0.237]. This could be attributed to an observation that was made that most HIV patients started attending ART clinics when their CD4 counts were below the cut-off points to be considered eligible for treatment. Such patients were often sick and frail and at such level of immunosuppression without COT, it is not surprising they would be exposed to an increased risk to malaria. The study could not adjust for protective effect of use of COT prophylaxes since almost all the patients took COT.

Fever is the most widely recognized manifestation of malaria. Fever may also be caused by HIV itself, opportunistic infections, or adverse drug reaction [26-28]. Several studies of the causes of fever in HIV-infected persons living in regions of stable malaria transmission have found that the majority of episodes of fever are not caused by malaria [28-30]. In this study, malaria was over-diagnosed based on the presence of fever (OR = 4.11). Since malaria was over-diagnosed based on fever, HIV patients were likely to have other illnesses misdiagnosed and treated as malaria [29].

It is also possible that the diagnostic practices adopted by healthcare providers in the various facilities could also account for the low confirmed cases of malaria. Most patients who reported with suspected malaria were not referred to the laboratory for confirmation of the diagnosis but were presumptively treated. Data for the proportions of laboratory confirmations made with either blood slide reading or RDT were not available.

Patients registered in the facilities were mainly referred through the Prevention of Mother-To-Child Transmission (PMTCT) programme and Voluntary Counselling and Testing (VCT). Observation from the sites revealed that men were more likely to hide their HIV status since they could not stand the stigma from the community they lived. Most of the men apart from the diagnostic processes were identified and registered after they were invited to the facilities during counselling of their spouses before administering their ARTs. This could account for the big difference in sexes of the patients’ folders that were randomly selected for review.

### Limitations of study

There was the possibility of sampling bias. Counterpart records should have been reviewed among HIV negative patients. Time was not weighted in the analysis. Evaluation for the records of all participants were carried out over a one year review period without considering rainy versus dry seasons. The number of positive cases were not enough to enable such analyses.

Another major limitation identified in this study was the high levels of missing data. There were about 3.5% of patients’ initial CD4 count missing and 32.9% of patients’ fever records, due to poor record keeping. There was also the possibility that there were other cases of malaria among the patients which had not been reported to the hospital and therefore did not appear in their records because they might have sought for treatment from drug sellers. The introduction of the National Health Insurance Scheme and the provision of free medical care for HIV patients in health facilities could have led to an increase in hospital attendance [31,32].

## Conclusions

The prevalence of confirmed malaria compared to patients managed presumptively for cases of malaria among HIV seropositive patients from the selected sites were 4.4% and 38.1% respectively. The study revealed an over-diagnosis of malaria based on reports of fever raising the possibility that other conditions which present with fever might go untreated. With the introduction of RDTs in many health facilities, confirmation of malaria before treatment should be encouraged at all levels of patient management.

## Competing interests

The authors declare that they have no competing interests.

## Authors’ contributions

The proposal was written by DAG, FB, CIF, KP and AM. SOA and GA were involved in the planning of the study. DAG, CIF, FB implemented the study with supervision from, CIF, FB, KP, SOA and JP. DAG collected the data. GA, EM, CIF, JP, FB and AM contributed to the data management, analysis and presentation. DAG, CIF, FB, DK and SN wrote the initial manuscript. All authors finally approved of the manuscript.
